# Supplementary material for: A library of chemically defined human N-glycans synthesized from microbial oligosaccharide precursors
Source: Sci Rep. 2017 Nov 21;7:15907. doi: 10.1038/s41598-017-15891-8 (PMC5698433; doi:10.1038/s41598-017-15891-8)
Supplement: Supplementary file 1 — Supplementary Information [file 41598_2017_15891_MOESM1_ESM.pdf]

Supplementary Information File

A library of chemically defined human *N*-glycans synthesized from microbial oligosaccharide precursors

Brian S. Hamilton, Joshua D. Wilson, Marina A. Shumakovich, Adam C. Fisher, James C. Brooks, Alyssa Pontes, Radnaa Naran, Christian Heiss, Chao Gao, Robert Kardish, Jamie Heimburg-Molinaro, Parastoo Azadi, Richard D. Cummings, Judith H. Merritt, and Matthew P. DeLisa

Supplementary Table 1. *N*-glycan structures produced in this study

Glycan Number	Symbolic Representation	Structure
2	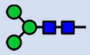	Manα1-6(Manα1-3)Manβ1-4GlcNAcβ1-4GlcNAc
3	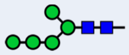	Manα1-6(Manα1-2Manα1-2Manα1-3)Manβ1-4GlcNAcβ1-4GlcNAc
4	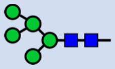	Manα1-6(Manα1-3)Manα1-6(Manα1-3)Manβ1-4GlcNAcβ1-4GlcNAc
5	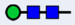	Manβ1-4GlcNAcβ1-4GlcNAc
6	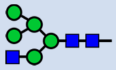	Manα1-6(Manα1-3)Manα1-6(GlcNAcβ1-2Manα1-3)Manβ1-4GlcNAcβ1-4GlcNAc
7	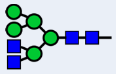	Manα1-6(Manα1-3)Manα1-6(GlcNAcβ1-2(GlcNAcβ1-4)Manα1-3)Manβ1-4GlcNAcβ1-4GlcNAc
8	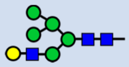	Manα1-6(Manα1-3)Manα1-6(Galβ1-4GlcNAcβ1-2Manα1-3)Manβ1-4GlcNAcβ1-4GlcNAc
9	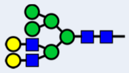	Manα1-6(Manα1-3)Manα1-6(Galβ1-4GlcNAcβ1-2(Galβ1-4GlcNAcβ1-4)Manα1-3)Manβ1-4GlcNAcβ1-4GlcNAc
10	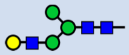	Manα1-6(Galβ1-4GlcNAcβ1-2Manα1-3)Manβ1-4GlcNAcβ1-4GlcNAc
11	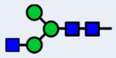	Manα1-6(GlcNAcβ1-2Manα1-3)Manβ1-4GlcNAcβ1-4GlcNAc
12	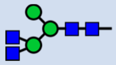	Manα1-6(GlcNAcβ1-2(GlcNAcβ1-4)Manα1-3)Manβ1-4GlcNAcβ1-4GlcNAc
13	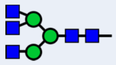	GlcNAcβ1-2(GlcNAcβ1-6)Manα1-6(GlcNAcβ1-2Manα1-3)Manβ1-4GlcNAcβ1-4GlcNAc
14	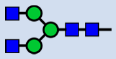	GlcNAcβ1-2Manα1-6(GlcNAcβ1-2Manα1-3)Manβ1-4GlcNAcβ1-4GlcNAc
15	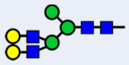	Manα1-6(Galβ1-4GlcNAcβ1-2(Galβ1-4GlcNAcβ1-4)Manα1-3)Manβ1-4GlcNAcβ1-4GlcNAc
16	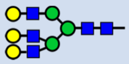	Galβ1-4GlcNAcβ1-2Manα1-6(Galβ1-4GlcNAcβ1-2(Galβ1-4GlcNAcβ1-4)Manα1-3)Manβ1-4GlcNAcβ1-4GlcNAc
17	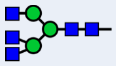	GlcNAcβ1-2Manα1-6(GlcNAcβ1-2(GlcNAcβ1-4)Manα1-3)Manβ1-4 GlcNAcβ1-4GlcNAc
18	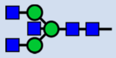	GlcNAcβ1-2Manα1-6(GlcNAcβ1-2Manα1-3)(GlcNAcβ1-4)Manβ1-4GlcNAcβ1-4GlcNAc
19	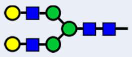	Galβ1-4GlcNAcβ1-2Manα1-6(Galβ1-4GlcNAcβ1-2Manα1-3)Manβ1-4GlcNAcβ1-4GlcNAc
20	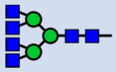	GlcNAcβ1-2(GlcNAcβ1-6)Manα1-6(GlcNAcβ1-2(GlcNAcβ1-4)Manα1-3)Manβ1-4GlcNAcβ1-4GlcNAc
21	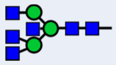	GlcNAcβ1-2Manα1-6(GlcNAcβ1-2(GlcNAcβ1-4)Manα1-3)(GlcNAcβ1-4)Manβ1-4GlcNAcβ1-4GlcNAc

Supplementary Table 2. Chemical shift assignments of the synthesized *N*-linked oligosaccharides

Residue	2 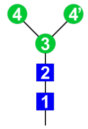	3 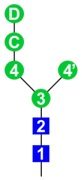	4 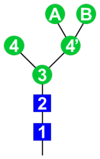	5 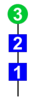	6 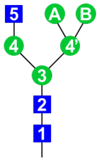	7 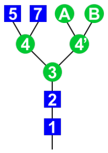	8 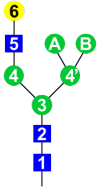	9 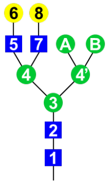	10 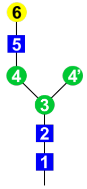	11 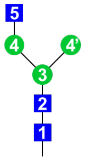
GlcNAc-1α	5.16	5.17	5.16	5.17	5.14	5.15	5.15	5.16	5.16	5.15
GlcNAc-1β	4.67	4.67	4.67	4.67	4.65	4.66	4.66	4.66	4.67	4.65
GlcNAc-2	4.58	4.58	4.57	4.57	4.55	4.56	4.55	4.56	4.58	4.57
Man-3	4.76	4.75	4.75	4.59	4.75	4.75	4.75	4.75	4.75	4.75
Man-4	5.08	5.32	5.06	-	5.08	5.07	5.08	5.08	5.07	5.08
Man-4’	4.89	4.89	4.84	-	4.84	4.84	4.84	4.84	4.87	4.88
Man-A	-	-	5.06	-	5.06	5.05	5.06	5.06	-	-
Man-B	-	-	4.88	-	4.88	4.87	4.88	4.88	-	-
Man-C	-	5.28	-	-	-	-	-	-	-	-
Man-D	-	5.02	-	-	-	-	-	-	-	-
GlcNAc-5	-	-	-	-	4.53	4.52	4.54	4.54	4.54	4.52
GlcNAc-5’	-	-	-	-	-	-	-	-	-	-
Gal-6	-	-	-	-	-	-	4.44	4.44	4.43	-
GlcNAc-7	-	-	-	-	-	4.50	-	4.52	-	-
Gal-8	-	-	-	-	-	-	-	4.43	-	-

Table 1. H1 chemical shifts. Glycans 2, 3, 5, 10-21 were analyzed at 25°C using a Varian Inova 600-MHz spectrometer (Complex Carbohydrate Research Center) with a cryoprobe. Glycans 4, and 6-9 were analyzed at 25°C using a Varian Inova 600-MHz spectrometer (Cornell) with a pulse field gradient probe. To calibrate spectra, HOD peak was set to δ_H = 4.77 ppm.

Supplementary Table 2. (continued)

Residue	12 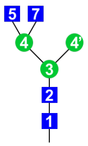	13 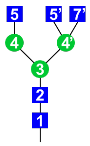	14 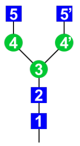	15 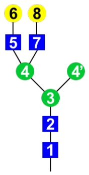	16 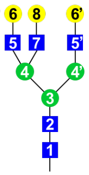	17 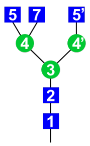	18 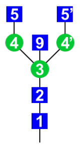	19 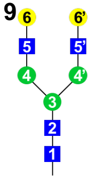	20 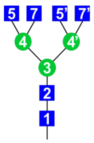	21 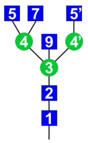
GlcNAc-1α	5.15	5.17	5.17	5.17	5.17	5.17	5.16	5.17	5.17	5.17
GlcNAc-1β	4.67	4.67	4.67	4.68	4.67	4.68	4.67	4.67	4.67	4.66
GlcNAc-2	4.57	4.58	4.57	4.58	4.58	4.58	4.58	4.59	4.58	4.58
Man-3	4.75	4.75	[1]n.d.	[1]n.d.	[1]n.d.	[1]n.d.	4.67	4.75	4.74	4.66
Man4	5.08	5.1	5.09	5.09	5.09	5.09	5.04	5.1	5.1	5.03
Man-4’	4.88	4.84	4.9	4.89	4.89	4.89	4.98	4.91	4.84	4.98
GlcNAc-5	4.52	4.54	4.54	4.55	4.53	4.53	4.53	4.56	4.55	4.5
GlcNAc-5’	-	4.51	4.54	4.53	4.57	4.51	4.53	4.56	4.54	4.53
Gal-6	-	-	-	4.45	4.44	-	-	4.45	-	-
Gal-6’	-	-	-	-	4.44	-	-	4.45	-	-
GlcNAc-7	4.49	-	-	-	4.51	4.54	-	-	4.51	4.53
GlcNAc-7’	-	4.54	-	-	-	-	-	-	4.51	-
Gal-8	-	-	-	4.43	4.45	-	-	-	-	-
GlcNAc-9	-	-	-	-	-	-	4.44	-	-	4.44

[1]n.d. - anomeric signal for Man3 overlaps with HOD peak at 25°C and is not visible in some proton spectra.

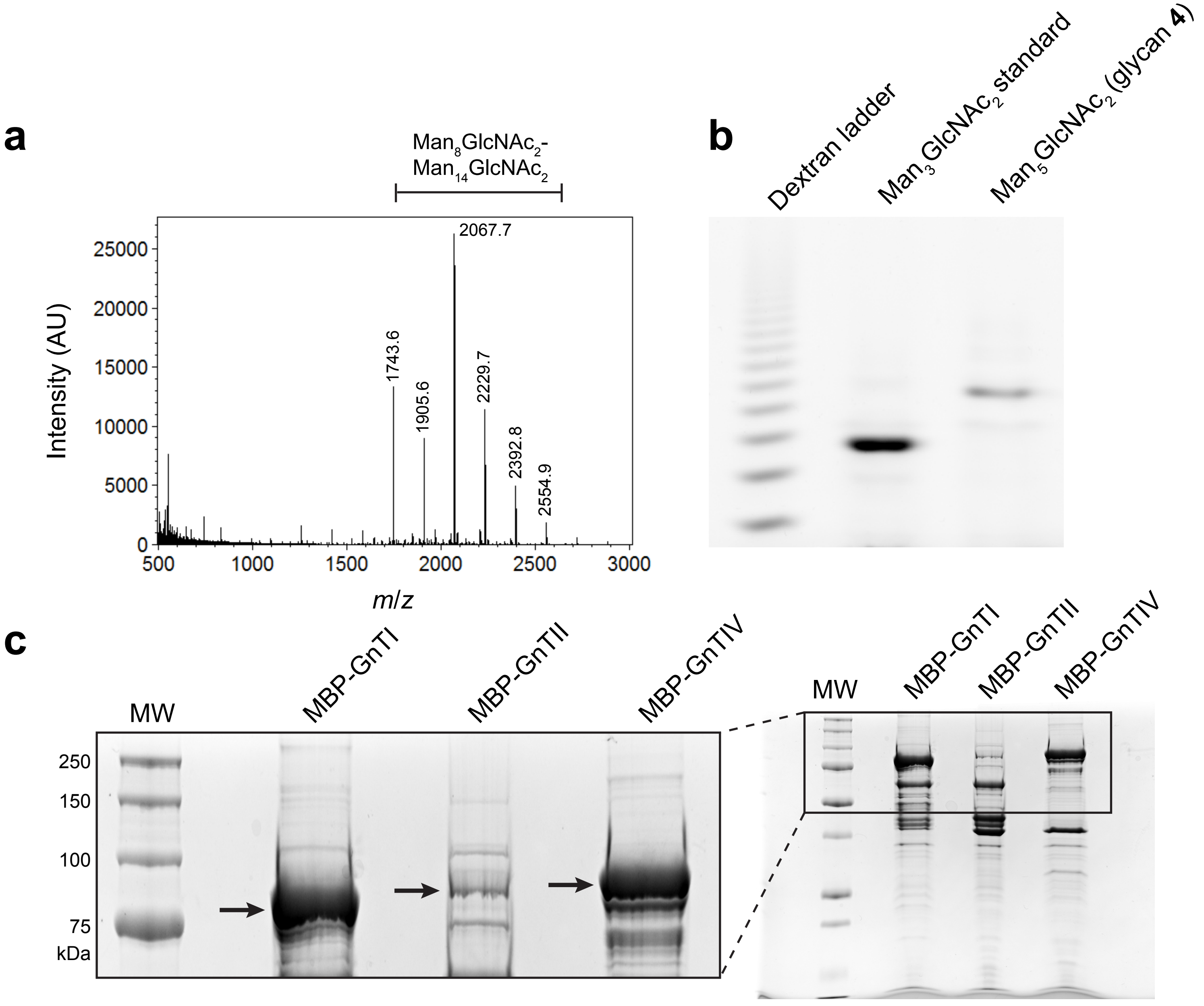

Supplementary Figure 1. MS and FACE analysis of yeast invertase-derived precursor oligosaccharides. (a) MALDI-TOF MS analysis of high-mannose oligosaccharides liberated from invertase by PNGase F. The predominant peak at 2067.7 *m*/*z* corresponds to $Man_{10}GlcNAc_2$. (b) FACE gel analysis of $Man_5GlcNAc_2$ produced by enzymatic deglycosylation of the oligosaccharide mixture shown in (a) demonstrates homogeneity and purity of glycan product. Commercially available $Man_3GlcNAc_2$ was used as a standard for quantification. (c) SDS-PAGE gel of fusion proteins comprised of the *E. coli* maltose-binding protein (MBP) and one of the following: *N. tabacum* GnTI, *H. sapiens* GnTII, or *B. taurus* GnTIV either GnTI, GnTII, or GnTIV, as indicated. All fusion proteins were expressed in *E. coli* Origami2(DE3) cells and purified by amylose affinity chromatography. Molecular weight (MW) ladder is shown at the left. Uncropped version of the SDS-PAGE gel is shown at the right, with the cropped section marked by the box.

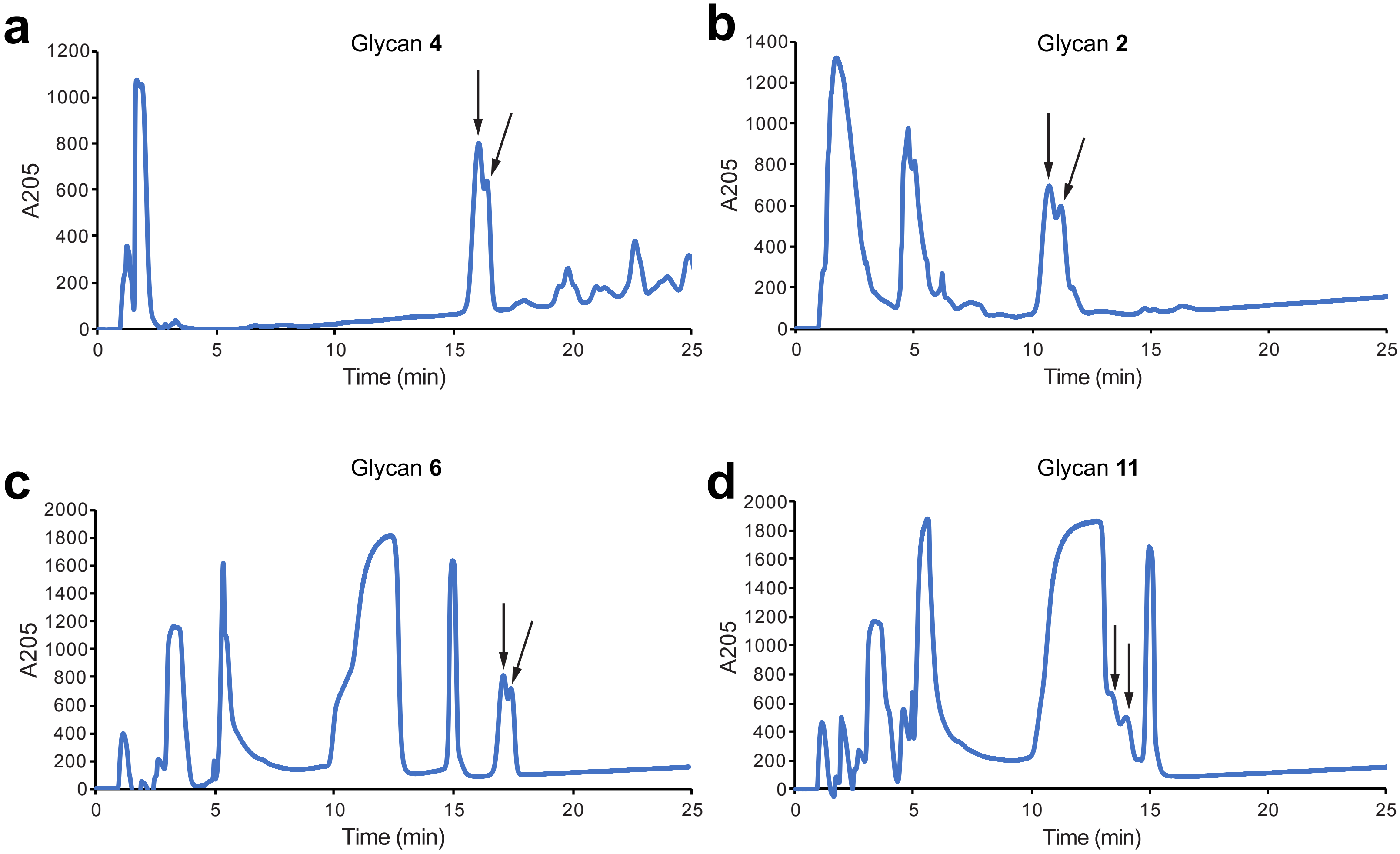

Supplementary Figure 2. Representative HPLC analysis of glycan precursors and products. (a) Partially purified glycan **4** (~150 mg isolated from invertase as described in the experimental procedures) was resuspended in 33% water, 67% acetonitrile, and resolved using a Waters XBridge BEH Amide column (130 Å, 5 μm, 4.6 mm X 100 mm). After an initial 5 min at 30% 20 mM ammonium formate in water, 70% acetonitrile, a linear gradient was run to 50% 20 mM ammonium formate in water, 50% acetonitrile over 20 min at room temp. Elution was monitored by absorbance at 205 nm, and fractions (~1.5 mL each) were analyzed by MALDI-TOF MS to verify the presence of glycan. This glycan eluted as a doublet with peaks at 16.0 and 16.4 min. Peaks eluting later in the gradient were high mannose contaminants as confirmed by MALDI-TOF MS. (b) Approximately 200 μg of dried glycan **2** (isolated from LLOs as described in the experimental procedures) was subjected to HPLC as described above. Glycan **2** eluted as a doublet, corresponding to alpha and beta anomers, with peaks at 10.7 and 11.2 min. (c) Glycan **6** was produced by subjecting HPLC-purified glycan **4** to an overnight reaction as described in the experimental procedures. The glycan **6** reaction was dried and subjected to HPLC as described above. This glycan eluted as a doublet with peaks at 17.1 and 17.4 min, making it well resolved from contaminant peaks and glycan **4**. (d) Glycan **11** was produced by subjecting HPLC-purified glycan **2** to an overnight reaction as described in the experimental procedures. The glycan **11** reaction was dried and subjected to HPLC as described for glycan **2**. Glycan **11** eluted as a doublet with peaks at 13.3 and 14.0 min. The shift in the elution time indicates addition of GlcNAc to glycan **2**; however, the assessment of precursor glycan **2** is prevented by overlap with the tail of a large peak corresponding to unreated UDP-GlcNAc. Doublet peaks corresponding to the glycan of interest are marked with arrows in each trace.

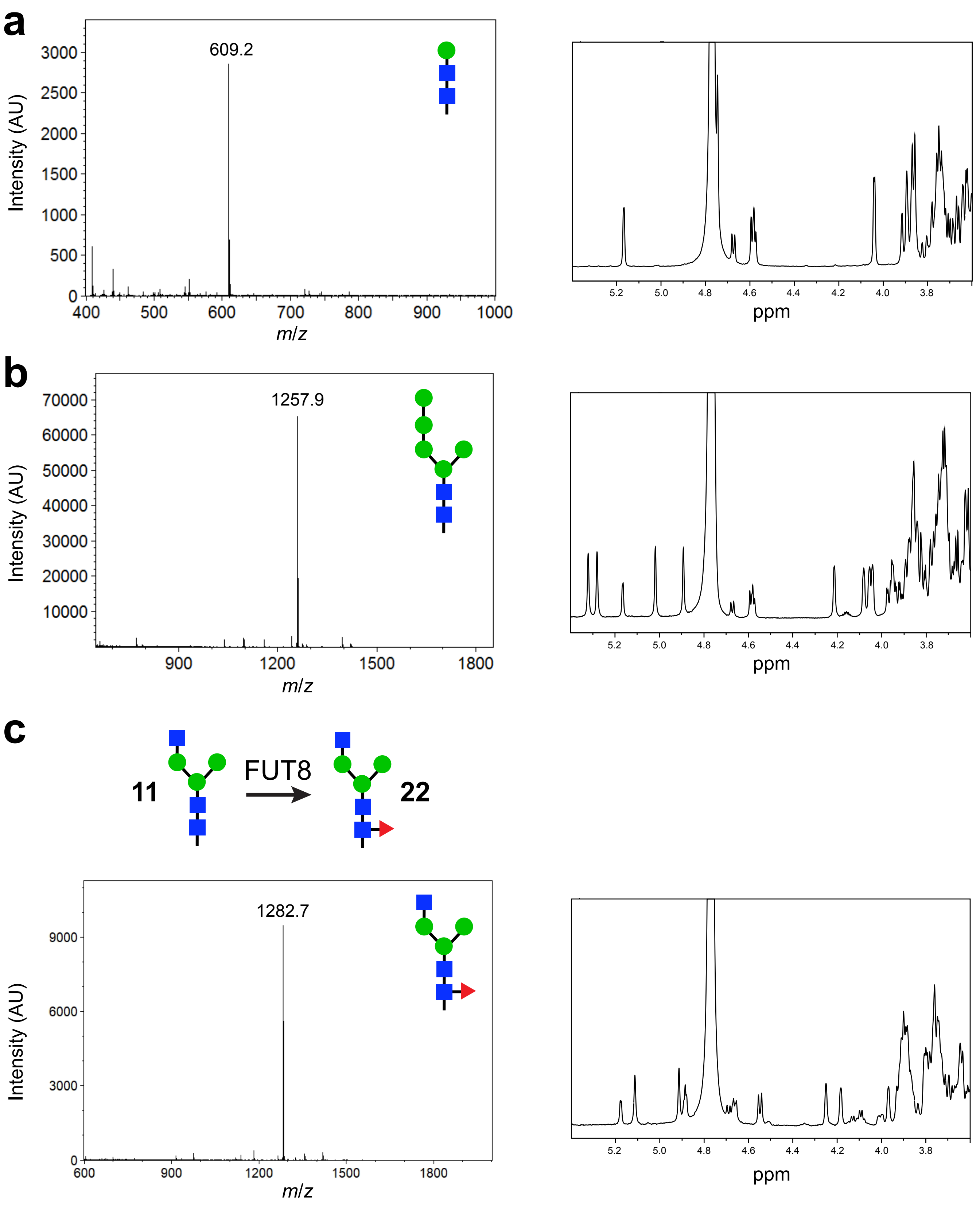

Supplementary Figure 3. Preparation of alternative precursor oligosaccharides for enzymatic remodeling. MALDI-TOF MS analysis (left panels) and 600-MHz ^{1}H NMR characterization (right panels) corresponding to: (a) $Man_1GlcNAc_2$ glycan synthesized by enzymatic deglycosylation of *S. cerevisiae* oligosaccharides; (b) the $Man_5GlcNAc_2$ glycan synthesized by glycoengineered *E. coli* cells; and (c) glycan 22, a fucosylated version of 11 generated by treatment with human FUT8 (R&D Systems), a commercially available α1,6-fucosyltransferase, in the presence of its natural donor substrate GDP-fucose.

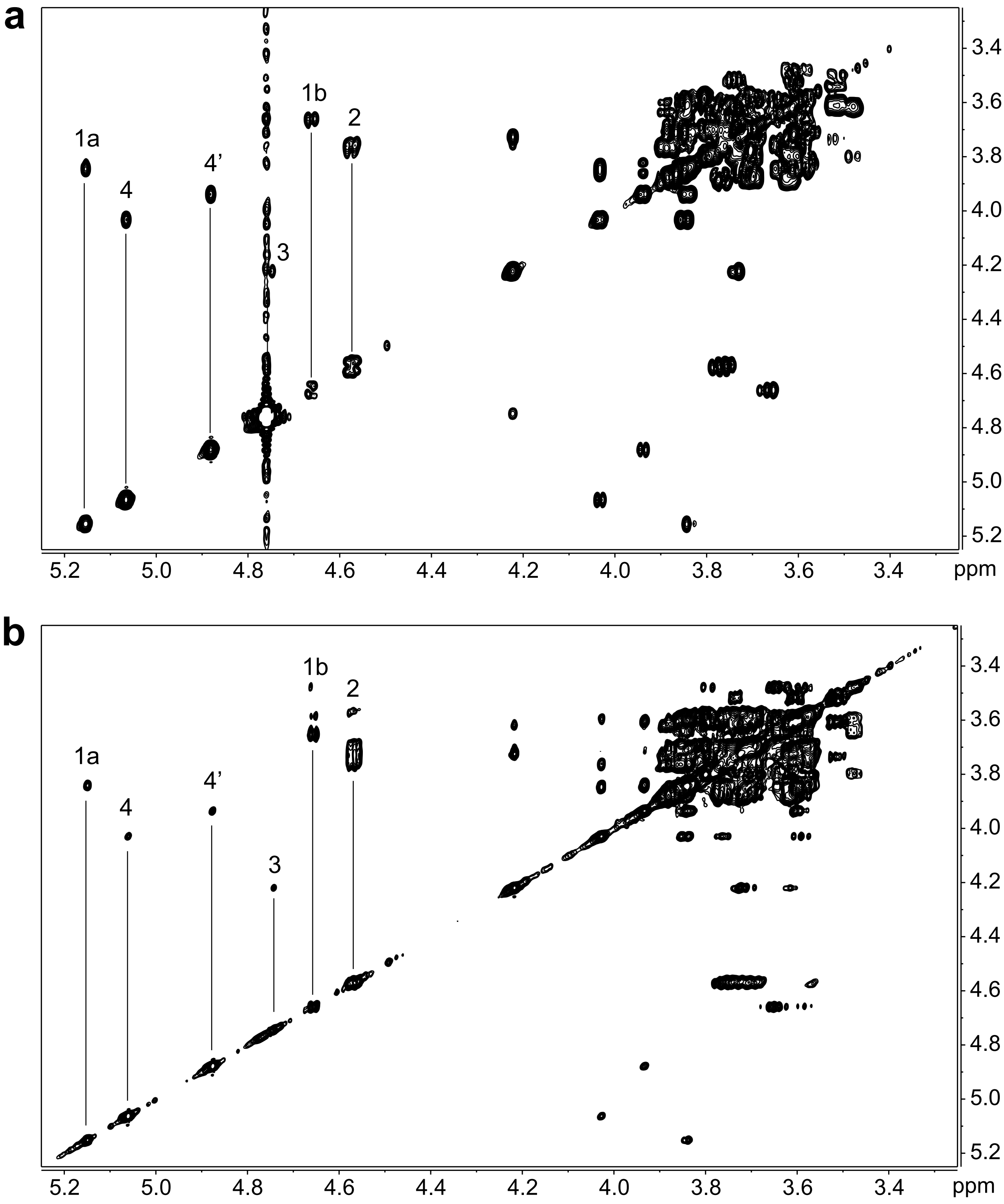

Supplementary Figure 4. (a) Partial ^{1}H-^{1}H-gradient COSY NMR spectrum of glycan **2**, acquired at 600 MHz and 25°C. Only the H1-H2-correlations are labeled for clarity. (b) Partial ^{1}H-^{1}H-zTOCSY NMR spectrum of glycan **2**, acquired at 600 MHz and 25°C. Only the correlations with H1 are labeled.

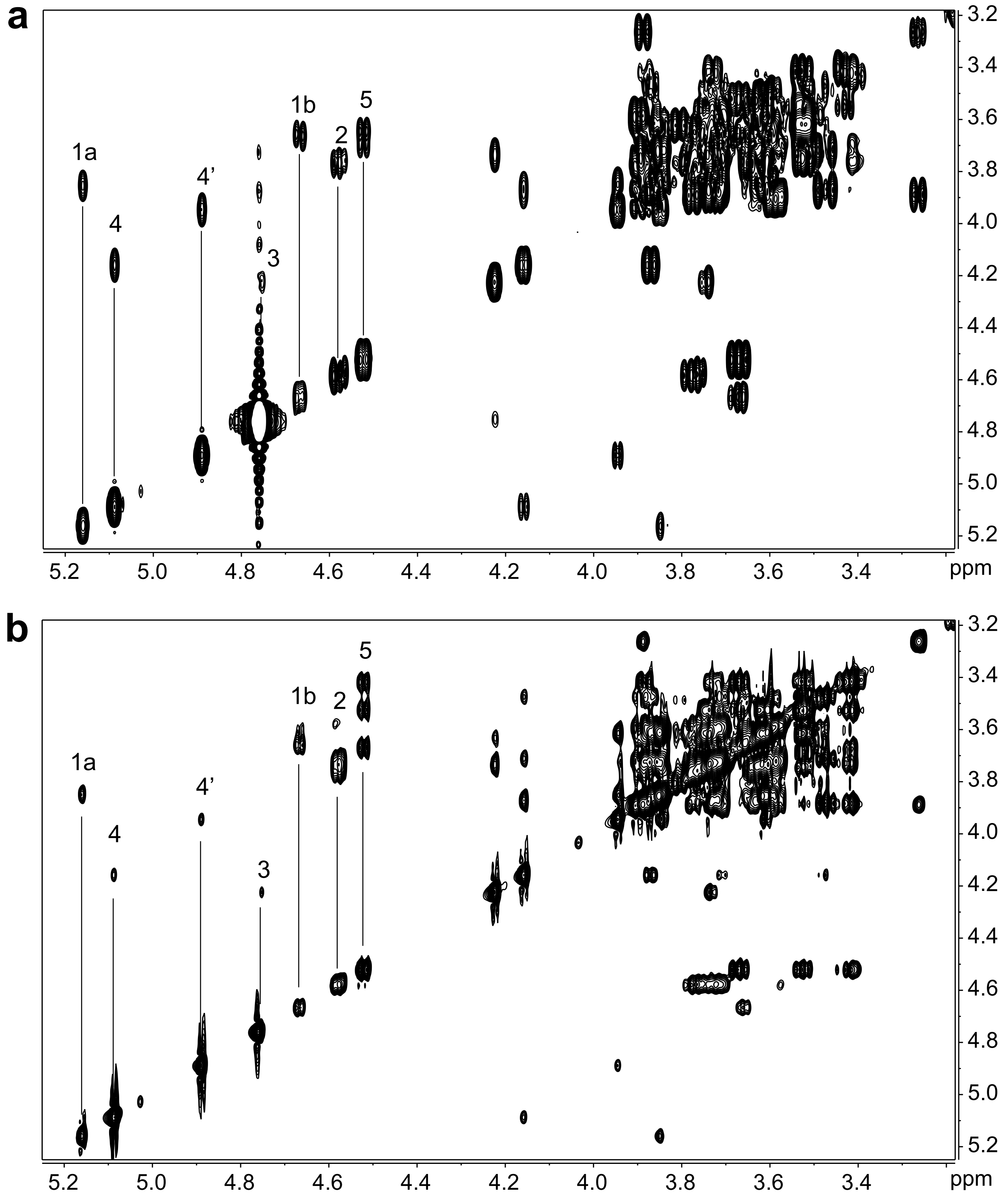

Supplementary Figure 5. (a) Partial ^{1}H-^{1}H-gradient COSY NMR spectrum of glycan **11**, acquired at 600 MHz and 25°C. Only the H1-H2-correlations are labeled. (b) Partial ^{1}H-^{1}H-zTOCSY NMR spectrum of glycan **11**, acquired at 600 MHz and 25°C. Only the correlations with H1 are labeled.

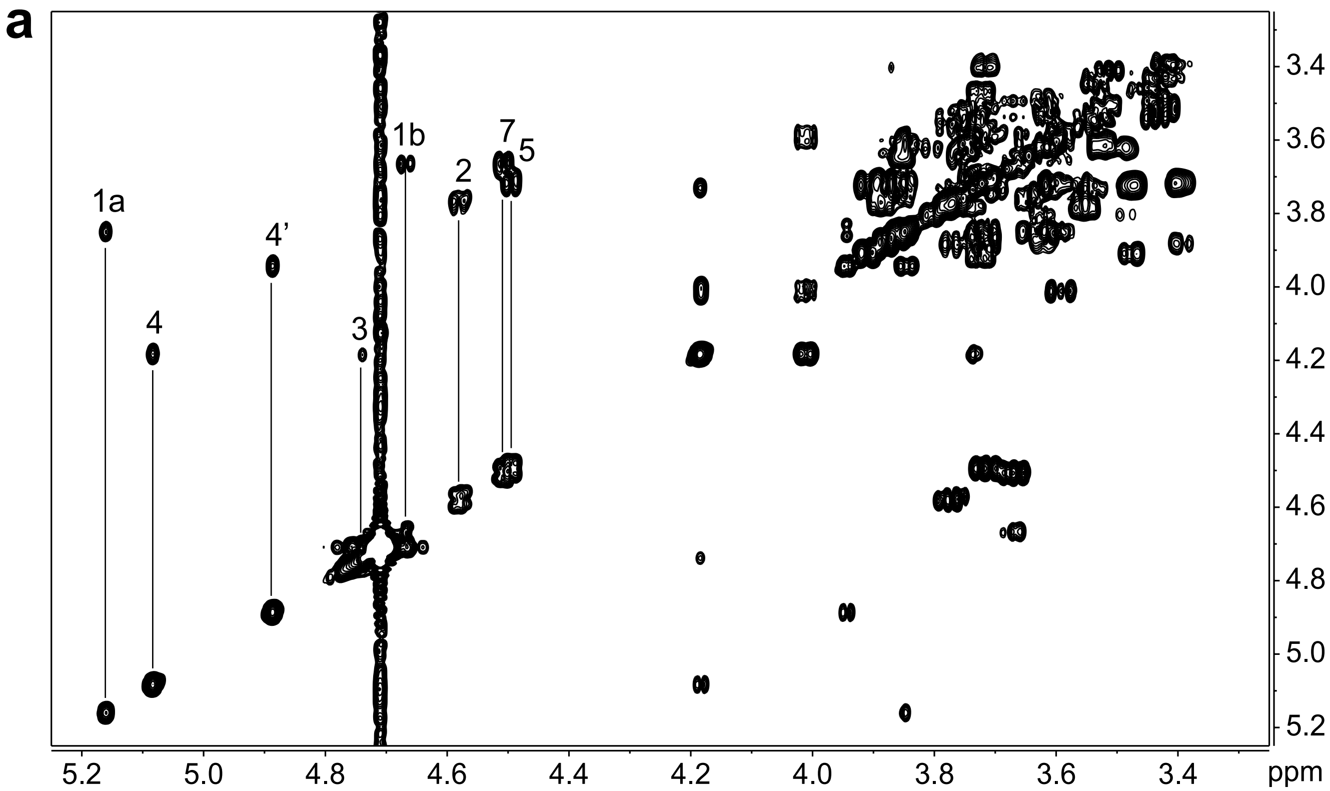

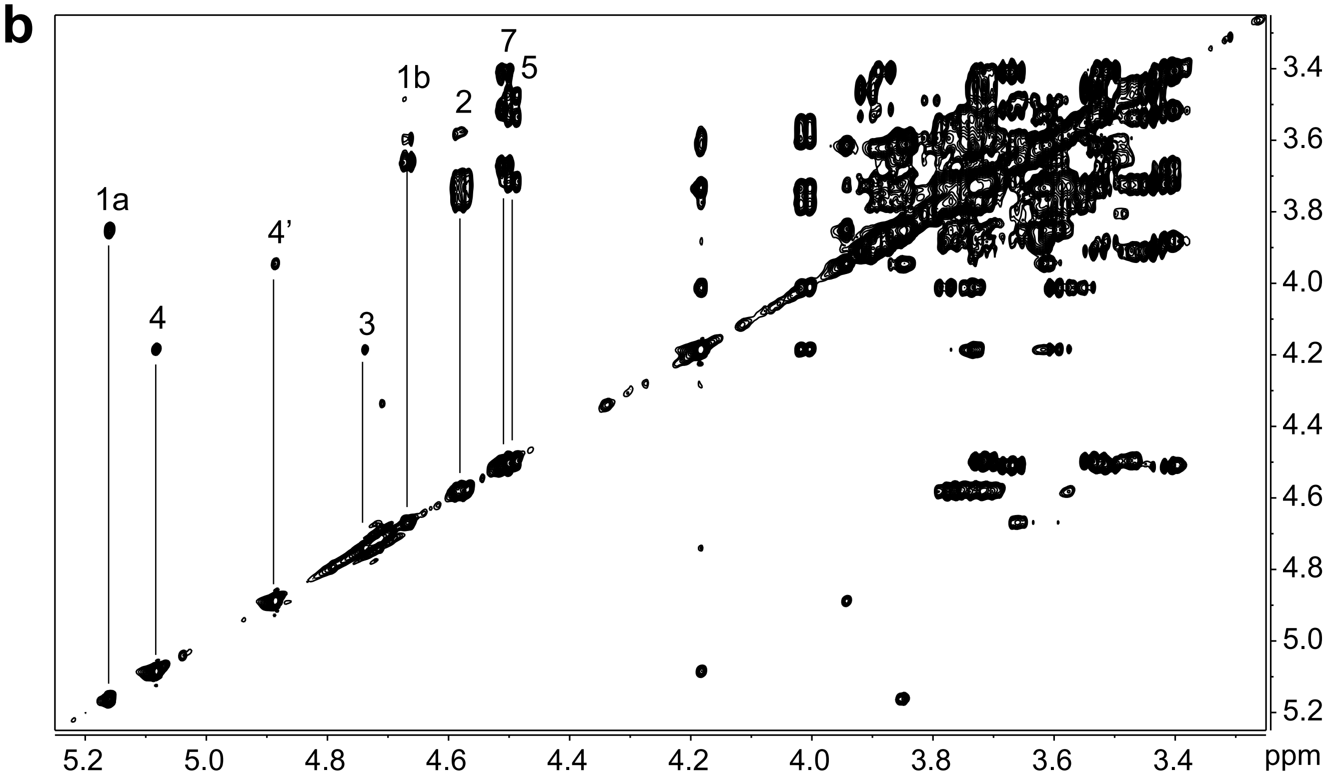

Supplementary Figure 6. (a) Partial ^{1}H-^{1}H-gradient COSY NMR spectrum of glycan **12**, acquired at 600 MHz and 30°C. Only the H1-H2-correlations are labeled. (b) Partial ^{1}H-^{1}H-zTOCSY NMR spectrum of glycan **12**, acquired at 600 MHz and 30°C. Only the correlations with H1 are labeled.

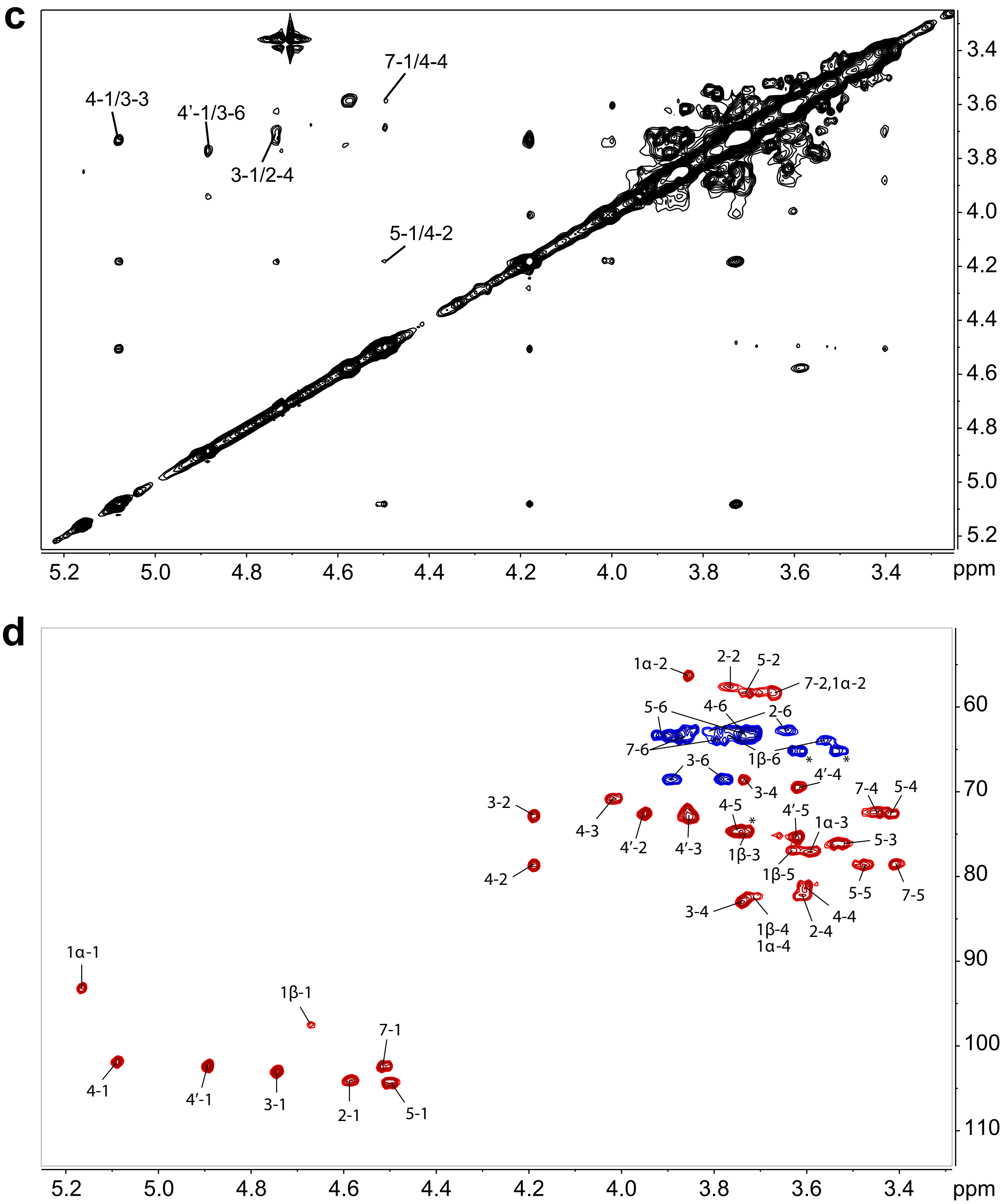

Supplementary Figure 6. (c) Partial ^{1}H-^{1}H-NOESY NMR spectrum of glycan **12**, acquired at 600 MHz and 30°C. Only the inter-residue correlations with H1 are labeled. (d) Partial ^{1}H-^{13}C-gradient HSQC NMR spectrum with multiplicity editing of glycan **12**, acquired at 600/150 MHz and 30°C. The signals labeled with asterisks are from contaminating glycerol.

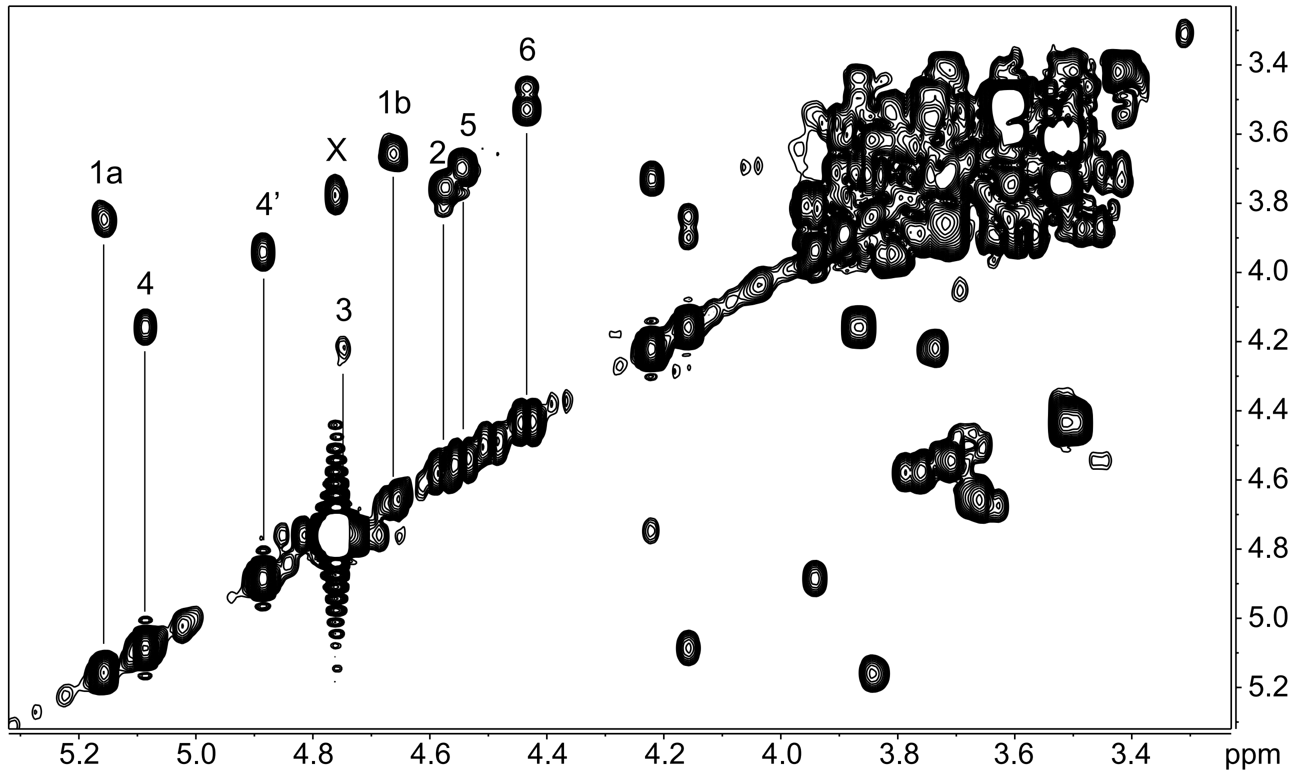

Supplementary Figure 7. Partial ^{1}H-^{1}H-gradient COSY NMR spectrum of glycan **10**, acquired at 600 MHz and 25°C. Only the H1-H2-correlations are labeled. The “X” marks an artifact, which is only present in the top half of the spectrum.
